# *Bacillus subtilis* HG-15, a Halotolerant Rhizoplane Bacterium, Promotes Growth and Salinity Tolerance in Wheat (*Triticum aestivum*)

**DOI:** 10.1155/2022/9506227

**Published:** 2022-05-07

**Authors:** Chao Ji, Huimei Tian, Xiaohui Wang, Xin Song, Ruicheng Ju, Huying Li, Qixiong Gao, Chaohui Li, Pengcheng Zhang, Jintai Li, Liping Hao, Changdong Wang, Yanyan Zhou, Ruiping Xu, Yue Liu, Jianfeng Du, Xunli Liu

**Affiliations:** ^1^College of Forestry, Shandong Agriculture University, Taian, China; ^2^College of Biological and Agricultural Engineering, Weifang University, Weifang, China; ^3^State Forestry and Grassland Administration Key Laboratory of Silviculture in Downstream Areas of the Yellow River, Shandong Agriculture University, Taian, China; ^4^College of Forestry, Shandong Agricultural University, Ecology Postdoctoral Mobile Station, Taian, China; ^5^Ministry of Agriculture Key Laboratory of Seaweed Fertilizers, Qingdao, China; ^6^Baiwo Biotechnology Co., Ltd., Linyi, China

## Abstract

Certain plant growth-promoting bacteria (PGPB) reduce salt stress damage in plants. *Bacillus subtilis* HG-15 is a halotolerant bacterium (able to withstand NaCl concentrations as high as 30%) isolated from the wheat rhizoplane in the Yellow River delta. A qualitative and quantitative investigation of the plant growth-promoting characteristics of this strain confirmed nitrogen fixation, potassium dissolution, ammonia, plant hormone, ACC deaminase, and proline production abilities. *B. subtilis* HG-15 colonization of wheat roots, stems, and leaves was examined via scanning electron microscopy, rep-PCR, and double antibiotic screening. After inoculation with the *B. subtilis* HG-15 strain, the pH (1.08–2.69%), electrical conductivity (3.17–11.48%), and Na^+^ (12.98–15.55%) concentrations of rhizosphere soil significantly decreased (*p* < 0.05). Under no-salt stress (0.15% NaCl), low-salt stress (0.25% NaCl), and high-salt stress (0.35% NaCl) conditions, this strain also significantly increased (*p* < 0.05) the dry weight (17.76%, 24.46%, and 9.31%), fresh weight (12.80%, 20.48%, and 7.43%), plant height (7.79%, 5.86%, and 13.13%), and root length (10.28%, 17.87%, and 48.95%). Our results indicated that *B. subtilis* HG-15 can effectively improve the growth of wheat and elicit induced systemic tolerance in these plants, thus showing its potential as a microbial inoculant that can protect wheat under salt stress conditions.

## 1. Introduction

Soil salinization is a serious abiotic stress that affects crop production, and approximately 20% of the world's cultivated land is currently under threat from salinization to varying degrees. As the fertile land available for cultivation decreases, soil salinization intensifies, owing to irrational irrigation and fertilization [1]. The Yellow River delta is undergoing the fastest salinization of any coastal ecosystem in the world. This area is abundant in light, is relatively cool, has highly saline soil, and is prone to drought, waterlogging, and alkalinization. Wheat is one of the most important cereals in the world and the main food crop grown in the Yellow River delta. As soil salinity increases, wheat growth and yield are threatened [2]. Reducing damage to wheat from long-term exposure to high salt concentrations and promoting the growth of wheat in low-salt concentration soils is a priority because there is a significant amount of coastal saline-alkali and secondary saline-alkali soil in this area.

Salt stress can destroy plants through secondary processes such as the promotion of ionic, osmotic, and oxidative stresses [3]. Na^+^ and Cl^–^ are the predominant salt ions in saline soil, and they are toxic to plants. Excessive accumulation of salt ions causes toxicity in plants and inhibits the absorption of other nutrient ions by plants, resulting in nutrient deficiency [4]. Soil salinization reduces the energetic state of rhizosphere soil water, causing the osmotic pressure of the soil solution to exceed the normal osmotic pressure in plant cells, which results in osmotic stress [5]. This leads to changes in the length, density, and structure of plant roots, reducing the uptake of soil water and nutrients, causing the plants to enter a state of physiological drought, and slowing or even killing the plant [6]. Oxidative stress causes damage to the cell membranes and photosynthetic system [7].

Previous studies have suggested that some beneficial soil microbes, including plant growth-promoting rhizobacteria (PGPR), rescue plant growth and yield under stress conditions. Antimicrobial substances and peroxidase may also be induced by plant growth-promoting bacteria (PGPB) to participate in plant defense responses and improve the resistance of plants to diseases and pests [8]. PGPB fix N, dissolve potassium and indole-3-acetic acid (IAA), promote iron carriers and 1-aminocyclopropane-1-carboxylic (ACC) deaminase (ACCD) activity, and stimulate other factors that directly affect plant growth. PGPB also secrete extracellular polysaccharides (EPS), osmotic regulators, and various volatile organic compounds (VOCs) that alter the structure and morphology of roots and elicit induced systemic tolerance (IST) in plants [9]. However, some PGPB tend to lose plant growth-promoting (PGP) traits with increased salinity in vitro [10]. Hence, the use of halotolerant PGPB that are selected based on both high salt tolerance and efficiency in expressing PGP traits may significantly advance our ability to grow crops in environments with natural or induced salinity. Few studies have screened PGPB with plant growth potential from the rhizoplane. In this study, a bacterial strain with high salt tolerance was isolated from the rhizoplane of wheat planted in saline-alkali soil. By testing PGP activity and colonization efficacy in wheat rhizospheres, we tested the relative efficacy of *Bacillus subtilis* strain HG-15 in protecting winter wheat from the harmful effects of soil salinity. To this end, soil pH and electrical conductivity (EC), rhizosphere soil nutrient and ion contents, wheat root and leaf development, photosynthesis, ion and nutrient uptake, oxidative damage, and other parameters were compared between inoculated and uninoculated wheat plants exposed to three different soil salt concentrations. The purpose of this study was as follows: (1) to screen and obtain plant-promoting bacteria that have high salt tolerance and can colonize saline-alkali soil, (2) to verify the effect of this bacterium on wheat growth and salt tolerance in representative highly alkaline soils in the Yellow River delta, and (3) to determine the effect of this strain on the chemical properties of wheat rhizosphere soil and the correlation between wheat rhizosphere soil and plant growth parameters.

## 2. Materials and Methods

### 2.1. Isolation of Bacteria

We isolated 12 strains of bacteria from root surfaces in the wheat rhizoplane in saline-alkali soil of the Yellow River delta in Shandong Province, China (118°49′15^″^E, 37°24′31^″^N). The soil parameters were as follows: pH, 8.535; salt content, 0.2143%; electrical conductivity (EC), 622 *μ*s/cm; organic matter, 25.847 g/kg; total N, 1.67 g/kg; and Olsen-P, 13.42 g/kg. Briefly, to isolate the bacterium, roots (1 g fresh weight) were thoroughly washed, homogenized in 0.5 × phosphate-buffered saline (PBS) (15 mL) [11], serially diluted to 10^−7^ in sterile nutrient agar (NA) medium with a 4% NaCl concentration, and placed in a 30°C incubator for 48–72 h [12]. The bacterium was subcultured twice. Finally, the isolates were streaked onto NA medium. A glycerol stock solution (30% *v*/*v*) of the isolate was prepared and stored at -80°C for later use. Based on colony morphology differences, 12 isolates were identified. Next, we measured the salt tolerance and other PGP activities of these strains to determine the strains with the most potential to promote plant growth in a pot experiment.

### 2.2. Screening for Salt Tolerance

The isolates were inoculated into Luria-Bertani (LB) medium (1% NaCl) and cultured for 24 h (30°C, 200 rpm/min, 8 × 10^8^ CFU/mL) as seed solutions. The seed solutions were inoculated, in triplicate, into LB medium with different NaCl concentrations (5%, 8%, 10%, 15%, 20%, 25%, and 35%) using a 2% inoculum. After 7 d, the absorbances of the cultures were measured with a TU-1810 spectrophotometer (Beijing Puxi General Instruments Co., Ltd., China) at 600 nm. At 30°C, 1 mL of each seed solution was absorbed and diluted to 10^4^ [12]. Uninoculated medium was used as a blank control. The isolates were tested for their ability to survive and tolerate salt in water-soluble fertilizer provided by Shandong Agricultural University Fertilizer Co., Ltd. The main components of potassium nitrate-containing humic acid water-soluble fertilizer products were as follows: macroelements, ≥400 g/L; total N, ≥360 g/L; potassium, ≥45 g/L; humic acid, ≥30 g/L; nitrate N, ≥90 g/L; ammonium N, ≥90 g/L; and amide N, ≥180 g/L. The main components of the solid water-soluble fertilizer (sulfuric ammonium yellow silver-humic acid-soluble fertilizer) were as follows: N-P_2_O_5_-K_2_O=17-5-23, macroelements, ≥45%; nitrate N, ≥ 9%; and fulvic acid, ≥ 3%. The fermentation broth of isolates was inoculated into fertilizer at 5% (*v*/*v*) and 5% (*w*/*v*) and placed at room temperature for 2 years. Based on the salt tolerance of the isolates in LB medium at different NaCl concentrations, we identified HG-15 as the strain with the highest salt tolerance in NA medium. A glycerol stock (15% *w*/*v*) of the isolate was prepared and stored at -80°C until further use.

### 2.3. Amplification and Sequencing of 16S rRNA Gene

To identify the bacterium at the molecular level, its 16S rRNA gene was amplified by PCR using a standard method [13]. The universal primers 27F (5′-AGAGTTTGATCMTGGCTCAG-3′) and 1492R (5′-TACGGYTACCTTGTTACGACT-3′) were used to amplify the 16S rDNA [14]. Amplified sequences were then gel purified using a TIANquick Midi Purification Kit (Tiangen Biotech, China) and sequenced. The obtained 16S rDNA sequence and sequences in NCBI were compared. Next, the pairwise evolutionary distance between the 16S rDNA sequence of the test strain and related bacterial strains was calculated, and a phylogenetic tree was constructed using the neighbor-joining method in MEGA software (version 5.0) [15]. Bootstrapping of 1000 replicates was used to assess clustering of associated taxa.

### 2.4. Bioassays for the Promotion of Growth and Enhancement of Salinity Tolerance Traits

#### 2.4.1. ACC Deaminase Assay

According to the method of Penrose and Glick [16], the strain was inoculated into 15 mL of tryptic soy broth medium and cultured for 24 h. The cells were harvested by centrifugation and washed with 0.1 M Tris-HCl (pH 7.6). We added 7.5 mL DF medium (3 mmol ACC as the only nitrogen source) and cultured the mixture overnight at 30°C. ACC deaminase activity was determined by measuring the amount of *α*-ketobutyric acid produced by the hydrolytic cleavage of ACC. The ACC deaminase activity of the strain was obtained by comparing the absorbance of the test sample with a standard curve of pure *α*-ketobutyrate (KB) and measuring the amount of KB at 540 nm.

#### 2.4.2. Osmolyte Accumulation

The proline content was calculated by comparison with a standard curve of pure proline. This method was modified from one described by Bates et al. [17] to determine the proline content. Glutamate acid can be extracted with a special reagent, and the results can be determined at 570 nm after adding a chromogenic agent. The reduced glutathione (GSH) concentration was determined at 412 nm using a kit (Solarbio, China).

#### 2.4.3. Screening for Other PGPB Traits

A Salkowski analysis was used to measure the IAA content in the strain after a 48 h incubation in liquid culture containing L-tryptophan (0.5 mg mL^−1^) [18]. The identification and quantitative analysis of abscisic acid (ABA), zeatin (ZA), salicylic acid (SA), jasmonic acid (JA), and gibberellin 3 (GA3) were performed following the protocols described in the S1. To evaluate the potential of nitrogen fixing of isolate, a preliminary test was conducted by culturing the isolate in Ashby's medium devoid of fixed N sources [19]. Bacterial N fixation efficiency was considered the mass of N (mg) fixed from the air per 1 g of carbohydrate consumed by the isolate and expressed as mg N/g sugar [20]. The strain was cultured in silicate solid medium. A clear area was formed around the colony, which indicated that the strain solubilized potassium feldspar [21]. Ammonia contents were estimated by the modified method of Singh and Jha [22]. Using yeast dextran as a substrate, the glucanase activity of a bacterial strain can be measured [23]. Cellulases are enzymes that breakdown cellulose into glucose and use sodium carboxymethyl cellulose as a substrate for cellulase activity [24]. The amount of reducing sugar in a reaction mixture can be determined using a dinitrosalicylic acid solution [25], and protease activity can be determined by measuring the tyrosine released by casein in a reaction mixture. Coomassie Brilliant Blue dye combines basic amino acids (especially arginine) and aromatic amino acid residues in proteins to change the maximum absorption peak of the dye from 465 nm to 595 nm, and the color of the solution changes from brownish black to blue. The absorbance value A595 measured at 595 nm is proportional to the protein concentration [26]. The activity of the iron-producing isolate carrier was determined using chrome azurol S, and growth of the isolate on CAS medium produced an orange halo that was believed to be due to the activity of an iron carrier [27].

VOCs produced by bacteria were determined by GCMS-TQ8050 (X). The mass spectra of unknown compounds were compared with those in NIST17 and NIST17s (National Institute of Standards and Technology) standard mass spectrometry libraries to determine the structure of the substances corresponding to the peaks.

The pathogenic fungi tested were *Fusarium oxysporum*, *Fusarium pseudograminearum*, *Rhizoctonia solani*, *Fusarium graminearum*, *Botryosphaeria ribis*, and *Botryosphaeria dothidea*. We inoculated pure pathogen cultures, applying each fungus (5 mm disk) to the center of a PDA plate (9 cm in diameter). After culture at 30°C for 24 h, the bacterial isolate was introduced into plates around the fungal cultures, with a distance between the isolate and the center of the dish of 2.5 cm. All plates were cultured in a constant temperature incubator at 30°C for 72 h, and the experiment was repeated three times. The percent growth inhibition was calculated as follows: inhibition (%) = [1–(fungal growth/control growth)] × 100% [28]. Each experiment was performed in triplicate, and the results were expressed as the mean with standard deviation.

#### 2.4.4. Physiological and Biochemical Characterization

We used standard protocols for physiological and biochemical tests of our isolate, including Gram stain, starch agar, IMViC (indole, methyl red, Voges-Proskauer, and citrate utilization test), and catalase tests [29]. In addition, we used the BIOLOG identification system (BIOLOG Microstation, Biolog, Inc., Hayward, CA) for biochemical testing with different carbon sources. The strain was assessed with 71 carbon sources and 23 chemical susceptibility assays according to the BIOLOG instructions.

#### 2.4.5. Test of Colonization

Colonization of the strain was determined independently using three methods. (1) An HG-15 strain showing rifampicin and spectinomycin resistance was obtained, and the number of colonies in the wheat rhizosphere soil was evaluated on the 7th, 14th, 21st, and 28th days after inoculation. Colony forming units (CFU) per gram of soil were determined using a method previously described by Islam et al. [30]. Each treatment was replicated thrice, and the experiment was repeated thrice. (2) The surfaces and cross sections of wheat roots, stems, and leaves were observed via scanning electron microscopy. HG-15 colonization was compared with that on the uninoculated control. (3) According to Singh and Jha [22], rep-PCR gene fingerprints of the HG-15 strain and strains recovered from wheat rhizosphere soil, roots, stems, and leaves indicate colonization. (4) The surface of wheat was sonicated, treated with 75% alcohol for 2 min and 2% sodium hypochlorite for 10 min for disinfection, and finally washed five times with sterile water. Treated roots, stems, and leaves were cut separately into small pieces of approximately 0.5 cm, and tissue sections were brought into contact with PDA medium containing rifampicin and spectinomycin. The water in the final wash step was coated to test the thoroughness of sterilization. Culture was carried out at 30°C for 3–6 d; treatment with the HG-15 strain was used as the control. HG-15 colonization of wheat roots, stems, and leaves was detected.

#### 2.4.6. Pot Experiments

The soil used for potted plants was obtained from the 0–20 cm soil depth in wheat fields in the Yellow River delta (118°41′07′′E, 37°17′17′′N) (Dongying City, Shandong, China) in October 2018. The soil was brought to the greenhouse and passed through a 0.5 cm sieve. The number of bacteria cultured from the sample was 1.36 × 10^4^ CFU·g^−1^ of dry weight soil. The upper and lower inner diameters of the flowerpot were 16.5 cm and 12 cm, respectively. The soil in each pot weighed 2.2 kg. Various soil parameters were as follows: pH, 8.329; salt content, 0.1492%; EC, 456 *μ*s/cm; organic matter, 23.51 g/kg; total N, 1.072 g/kg; Olsen-P, 0.0104 g/kg; K^+^, 0.6782 g/kg; Na^+^, 1.0162 g/kg; Ca^2+^, 0.23863 g/kg; and Mg^2+^, 0.50805 g/kg.

The HG-15 strain was inoculated in LB liquid medium at 200 rpm and 30°C for 12 h (logarithmic growth period). The bacterial suspension was then centrifuged at 1073 × *g* for 10 min to harvest cells. The HG-15 suspension (1 × 10^8^ CFU/mL) was adjusted to its final concentration with sterile water. The experimental wheat variety used was Jimai 21 (provided by the College of Agriculture, Shandong Agricultural University). The wheat seed surfaces were sterilized with 70% ethanol (*v*/*v*) for 2 min and washed with disinfectant water three times. Seeds were then placed in 1% (*w*/*v*) NaClO solution for 3 min and rinsed with sterile water three times to remove residual sodium hypochlorite [22]. A total of 72 pots were planted with 10 wheat seedlings in each pot. After 7 d, the aboveground height of wheat was approximately 5 cm, with wheat with taller or shorter heights removed. In the treatment group, 20 mL of cell suspension was applied; in the control group, 20 mL of water was used instead. Plants were irrigated with NaCl solution for salt stress (0.15% 456 *μ*s/cm, 0.25% 722 *μ*s/cm, and 0.35% 972 *μ*s/cm). At each concentration, 12 pots were either inoculated or not with the bacterium. The pots were placed in a completely random design, with each treatment repeated three times. The position of the pot was changed randomly during the trial to eliminate environmental errors. The root+shoot weight was considered the fresh weight of each plant. The dry weight of the plant was measured after placing a plant in the oven at 70°C for 2 d. For accuracy and precision, each sample was tested in triplicate. The growth of plants was measured in terms of root length, plant height, fresh weight, and dry weight.

#### 2.4.7. Effect of HG-15 on Soil Chemical Properties under NaCl Stress Conditions

Soil pH and EC values were analyzed with 1 : 2.5 and 1 : 5 soil-water ratios using digital pH (FE20) and EC (FE930) meters (Mettler Toledo, Switzerland), respectively. The organic carbon content was determined using 1 N potassium dichromate for titration and 0.5 N ferrous ammonium sulfate for back titration [31]. Olsen-P was extracted with 0.5 M NaHCO_3_ and determined according to the procedure described by Olsen [32]. Total N was measured using the Bremner [33] method.

Bulk soil was shaken off by uprooting the wheat. Soil still adhering to the root surface (concentrated within 2 mm of the root surface) was considered rhizosphere soil. We used a sterilized brush to collect the soil in a sterile bag on an ultraclean worktable. For ion analysis, 0.2 g of wheat rhizosphere soil was treated with 1 mL deionized water and 5 mL concentrated sulfuric acid overnight, and then, the cooked liquid was diluted in 50 mL of water. Measurements were carried out on 1 mL of the solution, which was extracted and diluted 10 times. Na^+^, K^+^, Ca^2+^, and Mg^2+^ contents were measured via inductively coupled plasma optical emission spectroscopy (ICP, Thermo Scientific™ iCAP™ 7000 Plus, USA) [22].

### 2.5. Effects of HG-15 on Plant Growth under NaCl Stress Conditions

#### 2.5.1. Photosynthetic Characteristics and Soluble Sugar and Proline Contents

Fresh leaves (1 g) were treated overnight with absolute ethanol, and then chlorophyll (Chl) and carotenoids were extracted and measured spectrophotometrically using the method described by Arnon [34]. First, 10 mL distilled water was added to 0.2 g dry leaves, the mixture was boiled for 30 min, and then, total soluble sugar (TSS) was quantified via the method of Thomas [35]. Proline content was determined according to the method described by Bates et al. [17], wherein valine was extracted with 3% sulfosalicylic acid and filtered. Next, an aliquot of the filtrate was supplemented with 1 mL ninhydrin and glacial acetic acid reagent. The mixture was then boiled for 1 h and placed on ice to stop the reaction, and the absorbance of the sample was measured at 520 nm using a UV spectrophotometer.

The net photosynthetic rate (A), stomatal conductance (gsw), intercellular CO_2_ concentration (Ci), and transpiration rate (E) of fully expanded leaves were analyzed using a LI-6800XT (Li-Cor, USA) portable photosynthetic apparatus after 28 d of treatment. Following an assessment of photosynthetic characteristics, chlorophyll fluorescence parameters were determined using an IMAGING-PAM (Walz, Effeltrich, Germany) fluorometer, and PSII primary light energy conversion efficiency was defined as Fv/Fm [36].

#### 2.5.2. Biochemical Analysis of Osmolytes in Plants after NaCl and Bacterial Inoculation

Approximately 0.2 g of fresh leaves was placed in a precooled mortar. First, 1 mL of 50 mmol/L buffer (containing 2% polyvinylpyrrolidone, pH 7.8, 4°C) was added, and the leaves were ground into a homogenate in an ice bath. Then, we washed the mortar with 0.5 mL of the above buffer solution to reach a final volume of 1.5 mL. After centrifugation at 12,000 × *g* and 4°C for 20 min, the supernatant was considered the crude enzyme solution. The mortar was rinsed with 0.5 mL of 50 mmol/L buffer (pH 7.8) to a final volume of 1.5 mL. The suspension was centrifuged at 12,000 × *g* at 4°C for 20 min. The supernatant served as the crude enzyme solution. Superoxide dismutase (SOD), peroxidase (POD), and catalase (CAT) activities were determined according to the method of Wu and von [37]. MDA was extracted from wheat leaves with thiobarbituric acid solution according to the method of Landi [38].

### 2.6. Statistical Analysis

Data analysis was performed using IBM SPSS 19.0. The Q-Q plot method was used to show that soil and plant parameters were normally distributed. For the same salt stress test with different treatments, *t* tests (*p* < 0.05) were used. One-way ANOVA and Dunnett's test were used to analyze the data under different salt stress conditions. A redundancy analysis (RDA) of soil and plant parameters was performed using Canoco 4.5.1 (Microcomputer Power, Ithaca, USA) software, and factors with significant explanatory functions were tested with conditional term effect analysis. Pearson's test (two-tailed) was used to analyze the correlation between soil and plant indexes, and the results were made into a heatmap using Origin 9.0 software.

## 3. Results

### 3.1. Isolation, Biochemical Characterization, and Identification of HG-15

**Figure 1:**
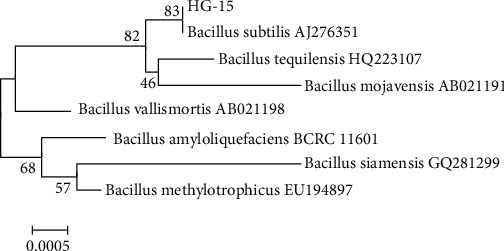
Phylogenetic tree showing relationships among bacteria associated with *B. subtilis* HG-15. The 16S rRNA gene sequence (1452 bp) of closely related strains was obtained from the NCBI GenBank database. The tree was generated with *n* = 1000 bootstraps using the neighbor-joining method in MEGA 5.0 software.

Based on colony morphology, 12 different bacterial isolates were recovered among the rhizoplane bacteria. Of these 12 isolates, the HG-15 strain with high salt tolerance (30% NaCl, *w*/*v*) was selected for further study. The biochemical and physiological characteristics of HG-15 are shown in Table 1. The 16S rDNA sequences of HG-15 were analyzed at the molecular level, where they were found to be most closely matched (99%) with 16S rDNA of *Bacillus subtilis* AJ276351 (Figure 1). The strain HG-15 sequence was deposited in the China General Microbial Culture Collection (CGMCC) under accession number 15773. The HG-15 16S rDNA sequence was deposited in the NCBI database under accession number MN689681.

### 3.2. Salt Tolerance and Plant Growth-Promoting Features

The test strain HG-15 exhibited a high salt tolerance and could survive in LB and NA medium with 30% (*w*/*v*) NaCl. After the strain was mixed with high-salt liquid hydrolyzed fertilizer and solid water-soluble fertilizer for 2 years, the strain recovery rates reached 82.63% and 85.37%, respectively. ACCD activity was 14.816 ± 0.965 *μ*mol/(mg·h), whereas IAA production was 154.53 ± 4.17 *μ*g/mL. The ABA, GA3, ZA, JA, and SA contents determined via HPLC at 3–7 d were 0.609 ± 0.026 *μ*g/mL, 0.103 ± 0.005 *μ*g/mL, 0.638 ± 0.014 *μ*g/mL, 0.430 ± 0.016 *μ*g/mL, and 3.865 ± 0.098 ng/mL, respectively. Other PGP characteristics were analyzed and are summarized in Table 2. The HG-15 strain inhibited *Fusarium oxysporum*, *Fusarium pseudograminearum*, *Rhizoctonia solani*, *Fusarium graminearum*, and *Botryosphaeria ribis*, but it did not inhibit *Botryosphaeria dothidea* (Figure S2).

### 3.3. Effects of HG-15 on Soil Chemical Properties under NaCl Stress Conditions

**Figure 2:**
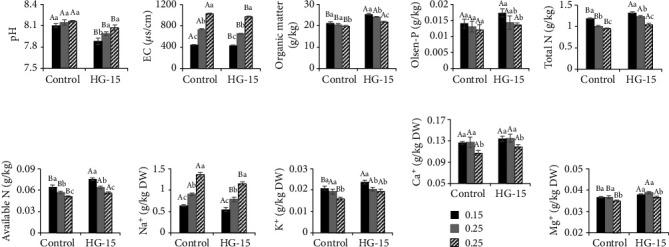
Effects of *B. subtilis* HG-15 inoculation on the physical and chemical properties of soil under different salinity conditions (0.15%, 0.25%, and 0.35% NaCl). (a) pH, (b) EC, (c) organic matter, (d) Olsen-P, (e) total N, (f) available N, (g) Na^+^, (h) K^+^, (i) Ca^2+^, and (j) Mg^2+^. Data are shown as the mean ± standard deviation (*n* = 5). Capital letters indicate the difference between CK (noninoculated control) and HG-15 inoculation groups under the same salt concentration (Student's *t* test, *p* < 0.05), whereas lowercase letters indicate differences under the three different salt concentrations in the control (or HG-15) group (one-way ANOVA, *p* < 0.05).

To evaluate the effects of HG-15 on wheat rhizosphere soil, we measured changes in pH, EC, and nutrient and metal elements in wheat rhizosphere soil. We then determined the growth of wheat in different soil salt concentrations. The rhizosphere soil pH decreased by 2.69%, 1.91%, and 1.08% at the 0.15%, 0.25%, and 0.25% NaCl salt concentrations, respectively (*p* < 0.05). Inoculation with HG-15 did not produce a significant difference in pH between soil with 0.25% and 0.35% salt concentrations. However, the pH of soil with a 0.15% salt concentration decreased significantly (Figure 2(a)). Compared with that of the uninoculated strains, the rhizosphere soil EC decreased by 3.17%, 11.48%, and 4.91% at 0.15%, 0.25%, and 0.25% NaCl salt concentrations, respectively (*p* < 0.05). The higher the salt concentration was, the higher the EC value (*p* < 0.05). The rhizosphere soil organic matter increased by 19.23%, 16.08%, and 10.08% at the 0.15%, 0.25%, and 0.25% NaCl salt concentrations, respectively (*p* < 0.05).

In rhizosphere soil inoculated with the HG-15 strain, there were significant differences in organic matter contents under the different salt concentrations (*p* < 0.05). Compared with that of the uninoculated strain, inoculation with the HG-15 strain did not significantly change the Olsen-P content in rhizosphere soil. In soil inoculated with the HG-15 strain, the Olsen-P content in rhizosphere soil with a salt concentration of 0.15% was higher than that in rhizosphere soil with a salt concentration of 0.35% (*p* < 0.05). In both the control group and the HG-15 group, the higher the salt concentration was, the lower the total N content (*p* < 0.05). Under the same salinity, the total N and available N contents in rhizosphere soil inoculated with HG-15 were higher than those in the control group (*p* < 0.05). Sodium ions, however, showed the opposite trend. Compared with that of the uninoculated strain, inoculation with the HG-15 strain increased the potassium content in the soil with concentrations of 0.15% (*p* < 0.05, 13.97%) and 0.35% (*p* < 0.05, 20.24%) but did not significantly affect soil at a 0.25% salt concentration. Inoculation with the HG-15 strain significantly increased the content of Mg ions in rhizosphere soil (*p* < 0.05) but did not affect Ca^2+^ content. Similarly, the Ca^2+^ and Mg^2+^ contents at a 0.35% salt concentration were significantly lower than those at 0.15% and 0.25% salt concentrations (Figure 2).

### 3.4. Effects of HG-15 on Plant Growth under Salt Stress

**Figure 3:**
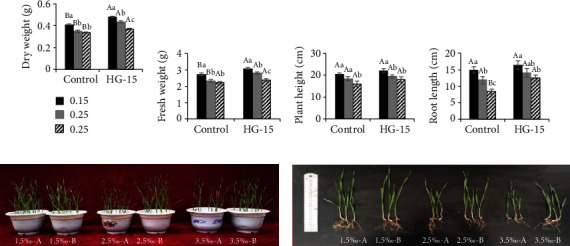
Effect of *B. subtilis* HG-15 inoculation on plant biomass under different salinity conditions (0.15%, 0.25%, and 0.35% NaCl). (a) Dry weight, (b) fresh weight, (c) plant height, and (d) root length. Data are shown as the mean ± standard deviation (*n* = 5). Capital letters indicate the difference between CK and HG-15 inoculation under the same salt concentration (Student's *t* test, *p* < 0.05), whereas lowercase letters indicate differences under the three different salt concentrations in the control (or HG-15) group (one-way ANOVA, *p* < 0.05).

**Figure 4:**
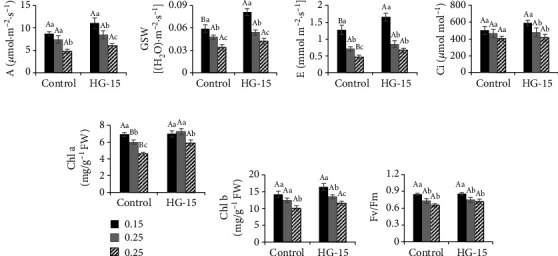
Effects of *B. subtilis* HG-15 inoculation on the chlorophyll content and photosynthesis of plants under different salinity conditions (0.15%, 0.25%, and 0.35% NaCl). (a) Chlorophyll A, (b) chlorophyll B, (c) net photosynthetic rate (A), (d) stomatal conductance (gsw), (e) transpiration rate (E), (f) intercellular CO_2_ concentration (Ci), and (g) Fv/Fm. Data are shown as the mean ± standard deviation (*n* = 5). Capital letters indicate the difference between CK and HG-15 inoculation under the same salt concentration (Student's *t* test, *p* < 0.05), whereas lowercase letters indicate differences under the three different salt concentrations in the control (or HG-15) group (one-way ANOVA, *p* < 0.05).

To study the effects of HG-15 on wheat growth under stress caused by different salt concentrations, we tested the biomass, photosynthesis, chlorophyll content, and chlorophyll fluorescence between HG-15-inoculated and noninoculated wheat seedlings after 28 d of growth. In the case of salt stress, the biomass (Figure 3), photosynthesis, chlorophyll content, and chlorophyll fluorescence levels (Figure 4) of the noninoculated seedlings were inhibited to various degrees. In the control group, there was no significant difference in dry weight and fresh weight at the 0.25% and 0.35% salt concentrations, but there was a significant difference after HG-15 inoculation. Compared with the uninoculated treatment, the dry weight (9.31–24.46%) and fresh weight (7.43–20.48%) of wheat inoculated with the HG-15 strain were significantly increased (*p* < 0.05). However, the plant height and root length were not significantly increased (Figure 3).

In the control group, soil with different salt concentrations had significant effects on the gsw, E, and Chl a of wheat (*p* < 0.05). After inoculation with the HG-15 strain, different salt concentrations had significant effects on wheat A, gsw, and Chl b (*p* < 0.05). The higher the salt concentration was, the lower the photosynthetic activity of wheat. At a 0.15% salt concentration, E (29.96%) was significantly increased in wheat inoculated with the HG-15 strain (*p* < 0.05). At a 0.25% salt concentration, the Chl a (20.24%) content in the leaves of wheat inoculated with the HG-15 strain was significantly increased (*p* < 0.05). At a 0.35% salt concentration, the E (43.65%) and Chl a (28.68%) contents increased significantly (*p* < 0.05) in wheat inoculated with the HG-15 strain (Figure 4).

### 3.5. Biochemical Analysis of Plants Treated with NaCl

**Figure 5:**
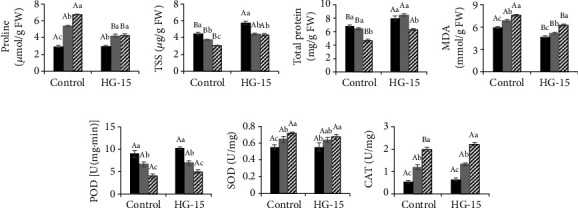
Effect of *B. subtilis* HG-15 inoculation on (a) proline, (b) TSS, (c) total protein, (d) MDA, (e) POD, (f) SOD, and (g) CAT under different NaCl salinity conditions. Data are shown as the mean ± standard deviation (*n* = 5). Capital letters indicate the difference between CK and HG-15 inoculation under the same salt concentration (Student's *t* test, *p* < 0.05), whereas lowercase letters indicate differences under the three different salt concentrations in the control (or HG-15) group (one-way ANOVA, *p* < 0.05).

The changes in certain chemical components, namely, proline, TSS, total protein, MDA, POD, SOD, and CAT, are known to play important protective roles during NaCl stress. This was assessed in wheat plants, with or without bacterial inoculation. After testing these components at different levels, we found that proline levels in uninoculated plants were increased under salt stress conditions of 0.25% and 0.35%, indicating that salt stress levels of wheat were reduced following inoculation with the HG-15 strain, with proline accumulation lower in HG-15-inoculated plants (Figure 5(a)). Salt stress also significantly reduced the TSS content in wheat. With increasing salt concentrations, the TSS content in wheat inoculated with the HG-15 strain was higher than that in uninoculated wheat, with increases of 29.31% (0.15%), followed by increases of 17.91% (0.25%) and 43.06% (0.35%) (*p* < 0.05) (Figure 5(b)). Compared to the control, salt stress also reduced the total protein content in uninoculated wheat by 16.36% (0.15%), 5.86% (0.25%), and 31.77% (0.35%), respectively (Figure 5(c)). The total protein content was higher in wheat inoculated with the HG-15 strain than in uninoculated wheat. The highest total protein content was observed in HG-15-inoculated plants under the 0.25% salt concentration, which showed a 31.48% increase over the uninoculated control (Figure 5(c)).

Lipid oxidation damage in wheat is reflected by an increased MDA content; therefore, MDA levels in wheat were estimated in relation to increased salt concentrations. The results indicated that the higher the salt concentration was, the higher the MDA content in uninoculated wheat (*p* < 0.05). Following inoculation with HG-15, the MDA contents at salt concentrations of 0.15%, 0.25%, and 0.35% were 21.63%, 24.34%, and 17.10% lower than those in uninoculated wheat, respectively (Figure 5(d)). However, inoculation of the HG-15 strain did not significantly affect the POD activity of wheat. The results indicated that the higher the salt concentration was, the lower the POD content in wheat (*p* < 0.05) (Figure 5(e)). Compared with plants treated with similar NaCl concentrations, inoculation with HG-15 did not significantly change the SOD activity in wheat. The results indicated that the higher the salt concentration was, the higher the SOD content in wheat (*p* < 0.05) (Figure 5(f)). The results of this study indicated that the CAT activity of wheat increased with an increase in salt concentration. Inoculation with bacteria increased the CAT activity of plants under salt stress, with a maximum increase of 18.38%, which was observed at a concentration of 0.15% NaCl. This increase became significant at 11.87% with a salt concentration of 0.35% (Figure 5(g)).

### 3.6. Root Colonization Assay

**Figure 6:**
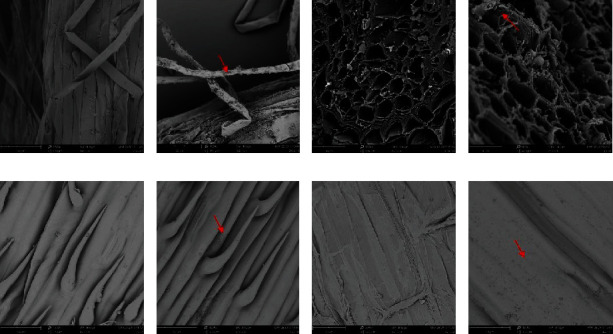
SEM micrographs of control- and +HG-15-treated 7-day-old wheat seedlings. (a and b) Root surface, (c and d) root cross section, (e and f) leaf surface, and (g and h) stem surface. The control (uninoculated) is shown on the left, and the HG-15-inoculated treatment (+HG-15) is shown on the right. Note the large number of bacterial colonies in the micrographs in the column.

The colonization efficiency of HG-15 in wheat roots, stems, leaves, and rhizosphere soil was determined by plate counts (with plates containing rifampicin and spectinomycin), rep-PCR measurement, and scanning electron microscopy. Changes in the number of bacteria following 7, 14, 21, and 28 d of rhizosphere colonization at different salt concentrations are shown (Table 3). The results showed that bacterial strain colonization changed the most from day 14 to day 21, and higher soil salt concentrations were associated with greater bacterial decreases. The lowest number of changes in bacterial strain colonization was observed between days 21 and 28, as reflected by the absence of a significant difference. The lower the soil salt content was, the higher the colonization number of the HG-15 strain (Table 3). Gene fingerprints of strains recovered from wheat roots, stems, and leaves and the HG-15 strain were compared using rep-PCR and found to be consistent, indicating that HG-15 can colonize wheat roots, stems, and leaves. The bacterial colonies and spore morphology of the isolated strains were similar, and no other strains with rifampicin resistance were detected in the soil; thus, there was no information for the other strains in Figure S1. Scanning electron microscopy also showed that HG-15 colonized wheat stems, leaves, and roots (Figure 6). The surfaces of treated roots, stems, and leaves were colonized as expected, but no obvious bacteria were found on the root, stem, or leaf surfaces of uninoculated control plants.

### 3.7. Relationship between Rhizosphere Soil and Plant Factors

**Figure 7:**
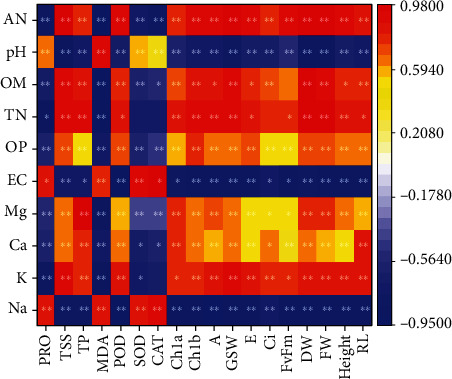
Correlation analysis of soil parameters and plant factors under the effect of strain HG-15. AN: available N; pH: soil pH; OM: organic matter; TN: total N; OP: Olsen-P; EC: electrical conductivity; Mg: Mg^2+^ concentration; Ca: Ca^2+^ concentration; K: K^+^ concentration; Na: Na^+^; PRO: proline; TSS: total soluble sugar; TP: total protein; MDA: malondialdehyde; POD: peroxidase; SOD: superoxide dismutase; CAT: catalase; Chl a: chlorophyll a; Chl b: chlorophyll b; A: net photosynthetic rate; GSW: stomatal conductance; E: transpiration rate; Ci: intercellular CO_2_ concentration; Fv/Fm: PSII primary conversion efficiency; DW: total dry weight of roots and shoots; FW: total fresh weight of roots and shoots; Height: plant height; RL: root length. Blue indicates a negative correlation between parameters, and red indicates a positive correlation between parameters. ^∗^*p* < 0.05 and ^∗∗^*p* < 0.01.

**Figure 8:**
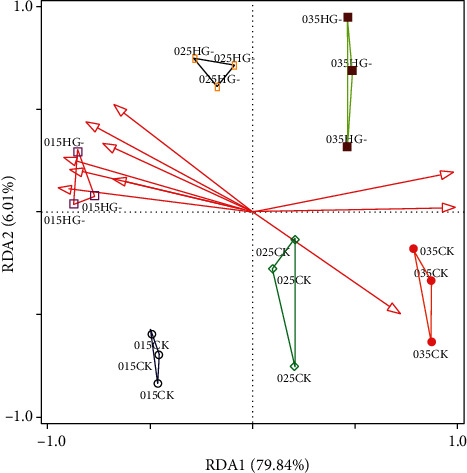
RDA of different soil parameters and plant parameters. 0.15CK: plant parameters under the 0.15% salt concentration; 0.25CK: plant parameters under the 0.25% salt concentration; 0.35CK: plant parameters under the 0.35% salt concentration; 0.15HG-: plant parameters after inoculation of strain HG-15 at a salt concentration of 0.15%; 0.25HG-: plant parameters after inoculation of strain HG-15 at a salt concentration of 0.25%; 0.35HG-: plant parameters after inoculation of strain HG-15 at a salt concentration of 0.35%; Mg: Mg^2+^ concentration; OM: organic matter; Ca: Ca^2+^ concentration; TN: total N; AN: available N; AP: Olsen-P; EC: electrical conductivity; Na: Na^+^; pH: soil pH.

We performed a correlation analysis of soil parameters and plant parameters under the effect of the HG-15 strain. As shown in Figure 7, changes in various soil factors can change plant proline, total sugar, total protein, MDA, POD, Chl a, Chl b, A, gsw, E, DW, FW, plant height, and root length (*p* < 0.05). Available N, pH, Olsen-P, Mg, and Na had significant effects on plant factors (*p* < 0.01). In addition, pH, EC, and Na showed a strong negative correlation with plant photosynthesis and biomass accumulation. We also found that these factors were positively correlated with proline, MDA, CAT, and SOD contents. According to the RDA, total N, organic matter, and available N and Mg^2+^ were strongly positively correlated with plant growth, whereas EC, Na, and pH were strongly negatively correlated with plant growth (Figure 8). Combined with the data in Table S1, we found the following: Na can explain 76.6% of the variation in plant growth, and OM, EC, and Ca^2+^ can explain 4.6%, 3.2%, and 2.2% of the variation in plant growth, respectively (*p* < 0.05). The results indicated that Na^+^ content has a significant negative correlation with plant parameters. The effects of OM, EC, and Ca^2+^ on plant parameters were positively correlated. The main effects and interactions of salinity and bacteria on the measured parameters are shown in Table S1. Salt had a major effect, whereas bacteria had no major effect on Olsen-P. Bacteria had a major effect, whereas salt did not have a major effect on parameters such as Ci, Fv/Fm, and SOD. The soil parameters that showed salt and bacterial interactions were pH, EC, organic matter, and total N. The plant parameters that showed salt and bacterial interactions were dry weight, fresh weight, plant height, root length, gsw, Chl a, proline, TSS, and total protein (Table 4). RDA accounted for 85.81% of the variation in the soil and plant parameters. The results of this study show that changes in soil parameters have a significant impact on plant parameters. Soil total N, organic matter, Mg, Ca, Olsen-P, and available N are positively correlated with plant parameters, whereas pH, EC, and Na are negatively correlated with plant growth.

## 4. Discussion

The microorganisms used in soil–plant systems enhance crop viability, yield, quality, and tolerance to abiotic stress and are therefore considered important agricultural biostimulants [39]. However, as several studies have shown, many microbial products are not always effective in the field [40, 41]. Under salt stress, many strains also lose their PGP characteristics [1]. We emphasize that the plant root surface is an important plant tissue for material exchange between plants and the external environment and direct contact with microorganisms. In this study, we measured the growth-promoting characteristics of *Bacillus subtilis* HG-15 (Table 2). These growth-promoting traits of halotolerant rhizoplane bacteria perhaps enhanced plant growth and biomass production in salinized plants under greenhouse conditions.

Several PGPB can synthesize the enzyme ACC deaminase, which converts the plant ethylene precursor ACC to ammonia and *α*-ketobutyrate, thus reducing the accumulation of ethylene in the plant and avoiding ethylene-mediated growth inhibition in response to abiotic stresses such as salinity [42]. ACC deaminase activity has also become one of the most important criteria for the screening of salt-tolerant PGPB [43]. The results of this study showed that the isolated salt-tolerant bacteria HG-15 significantly increased the dry weight and fresh weight of wheat, and the root length of wheat was significantly increased by the inoculation of HG-15 under the 0.35% salt concentration stress (*p* < 0.05) (Figure 3). We speculate that this is closely related to the production of ACC deaminase by the strain.

Research by Ahmad Y.N. et al. [44] showed that plants can respond to exogenous plant hormones, which can alleviate the adverse effects of salinity. In this study, the HG-15 strain had the ability to produce IAA, which is important to help maintain leaf growth and reduce plant productivity restrictions caused by saline-alkali stress. Inoculation with GA-producing PGPB can affect endogenous GA levels in plants [45] and help regulate plant leaf and root meristem size, cell division and elongation, and hypocotyl and stem growth [46, 47]. Increased growth of drought-stressed lettuce plants inoculated with a ZA-producing *B. subtilis* strain suggested modulation of root-to-shoot ZA signaling [48]. ABA also plays a crucial role in plant-PGPR interactions [49]. For example, in a previous study, following the inoculation of plants with ABA-producing strains such as *B. licheniformis* Rt4M10, *P. fluorescens* Rt6M10, and *A. brasilense* Sp 245, the internal ABA content increased, and inoculated plants became more resistant to drought than uninoculated plants [50]. The resulting changes in ABA levels may mitigate the plant's sensitivity to water scarcity. This study confirmed the promoting effect of the HG-15 strain with multiple PGP characteristics (Table 2) on wheat plant growth under salt stress. Inoculation with this bacterium also regulates nutrient enrichment and the Na^+^/K^+^ ratio in the plant rhizosphere soil, thereby protecting plants from salt damage. Although there is evidence that PGPB improves plant salt tolerance by changing the state of endogenous hormones, how PGPR affect this process is unknown. We speculate that the hormones produced by PGPB in vitro and the hormones produced in plants can be recognized and utilized by plants and directly affect the transmission of relevant signals in plants. Crucially, it is now rare to detect these hormones in the metabolites of a single strain (Table 2).

Increasing the activity of key enzymes in plants is an important antioxidant defense mechanism. Key enzymes, such as SOD and CAT, remove excess reactive oxygen species and protect plants from salt poisoning [30, 51]. These plants exhibited an accumulation of osmolytes (e.g., sugar and proline) and an increase in antioxidant enzyme activity (e.g., SOD, POD, CAT, and ascorbate peroxidase) compared to uninoculated plants (Figures 5(a)–5(c)). Mahdi et al. [52] also found that plants can maintain ion homeostasis and therefore accumulate less reactive oxygen species, which promotes plant growth, when inoculated with the bacteria *Klebsiella*, *Pseudomonas*, *Agrobacterium*, and *Ochrobactrum*. This is consistent with the results of this study (Figures 5(d)–5(g)).

Abiotic stress usually leads to decreases in leaf water content and affects the content of osmotic regulators [53]. Studies have shown that under abiotic stress, ABA can induce the accumulation of osmoregulators such as proline and soluble sugars [54]. Inoculation with ABA-producing PGPB often decreases the accumulation and concentration of ABA in roots and significantly alters the long-distance signaling of shoot-to-root ABA transport in the phloem and root-to-shoot ABA transport in the xylem [7, 51]; the resulting changes in ABA levels may mitigate the plant's sensitivity to water scarcity. PGPB promote the ability of plants to accumulate compatible solutes to maintain intracellular osmotic balance [55]. Inoculation with the HG-15 strain resulted in an increase in certain osmolytes, such as total soluble sugar and total protein content, and a decrease in malondialdehyde content. Especially in soil with the 0.25% and 0.35% salt concentrations, the TSS and total protein contents of wheat inoculated with the HG-15 strain were significantly increased (*p* < 0.05) (Figures 5(b) and 5(c)). In this study, it was found that *B. subtilis* HG-15 can produce osmotic adjustment factors such as ABA, proline, glutamic acid, and GSH. This improves the water use efficiency and salt tolerance of wheat [3]. Figure 8 shows that Mg, Ca, and K have a positive effect on the accumulation of TSS and total protein in plants and have a negative correlation with the accumulation of proline, while the result of Na ions was the opposite. This suggests that the inoculation of plants with PGPB alleviated NaCl stress; proline accumulation was lower in HG-15-inoculated wheat.

The interaction between microorganisms and plant roots is an important factor that promotes nutrient element circulation in soil. Organic matter accumulated by plant photosynthesis is released into the soil through plant roots, providing nutrients and energy for the growth of microorganisms, which decompose insoluble minerals in the soil through their metabolic activities and provide mineral nutrients for the growth of plants [56]. In this study, the HG-15 strain absorbed different amounts of Na, K, and Mg in soils with different salt concentrations (Figure 2). The effect of the HG-15 strain on the soil available P content was not significant. This may have been due not only to the high pH and strong buffering capacity of the soil tested but also to the limited amount of organic acids produced by the strain. Phosphorus plays an important role in the improvement of saline-alkali land. Moreover, better improvement of saline-alkali soil still requires the addition of nutrients such as external acid phosphate fertilizer.

The main function of K is to enhance the photosynthesis of plants, promote the synthesis of sugar, starch, and protein in plants, and improve the disease resistance of crops. Silicate bacteria can release soil potassium, promote the transfer of K from roots to leaves, and play an important role in promoting plant nutrient absorption and osmotic pressure regulation [57]. The HG-15 strain dissolves potassium, and the enrichment of K in rhizosphere soil greatly promotes the growth of plants. Excessive accumulation of Na^+^, which occurs under salt stress, leads to osmotic stress, which causes Na^+^/K^+^ imbalance or even ionic poisoning in plants [58]. As a result, it can decrease the intensity of root injury, cause stomata to close and reduce transpiration tension, and interfere with the ability of the plant body to absorb water, which inhibits its physiological activity and causes a decrease in the photosynthetic rate [59]. Eventually, the photosynthetic organs of plants are destroyed, and the photosynthetic rate is reduced [60]. Reduced Na^+^ contents and a higher K^+^/Na^+^ ratio leading to stress alleviation were also observed by Yasin et al. [39] in PGPR-inoculated *Capsicum annum* growing under salt stress. The results showed that photosynthesis was stronger in wheat inoculated with HG-15 than in wheat not inoculated with the strain (Figures 4(c) and 4(e)). We speculate that this is due to the PGPR changing the ion content in the rhizosphere of plants. The results in Figures 7 and 8 also confirm that K and Mg in rhizosphere soil have a positive effect on plant photosynthesis. Although HG-15 inoculation did not significantly affect Ca^2+^ in rhizosphere soil, through correlation analysis (Figure 7) and RDA (Figure 8), we can see that Ca^2+^ also has a positive effect on plant growth parameters. Unfortunately, the HG-15 strain failed to produce a significant increase in the Ca^2+^ concentration in rhizosphere soil.

From the results of RDA and correlation analysis, available N had the largest positive correlation with biomass accumulation and photosynthesis of plants. It can be inferred that the available nitrogen content in rhizosphere soil has the greatest influence on plant growth under salt stress. N is the nutrient that most restricts plant productivity. Salt stress can interfere with plant nitrogen nutrition and reduce the N content in plant tissues [61]. Nitrogen-fixing bacteria are an important source of available nitrogen for plants in saline soil. Moreover, nitrogen-fixing PGPB can maintain normal cell turgor and metabolism of plants by producing osmotic factors [62].

This study isolated a rhizoplane bacterium, *Bacillus subtilis* HG-15, with good growth-promoting properties and high salt tolerance (30% NaCl salt concentrations). Through a series of physiological and biochemical tests, scanning electron microscopy (SEM), rep-PCR, and double antibiotic screening techniques, we aimed to analyze the mechanisms of the growth-promoting abilities and the halotolerant properties of this strain from its genetic characteristics, the change in chemical properties of rhizosphere soil, and the improvement of the growth state of wheat. In particular, we detected that the pH of wheat rhizosphere soil inoculated with the HG-15 strain was significantly reduced (*p* < 0.05), which was not easy. We speculate that this is related to the highly effective inoculation of the strain and the excellent colonization ability of the strain. Since the strain was isolated from the root surface of the plant, we speculate that in addition to its stable colonizing ability inside the plant and the rhizosphere, it can also produce a large amount of acidic substances. Although these acids did not significantly change the amount of available phosphorus in the soil, they lowered the soil pH (Figure 2). Different salt-tolerant PGPRs have different PGP characteristics, and different characteristics will cause plants to respond differently [3]. This study confirmed the promoting effect of the HG-15 strain with various PGP characteristics (Table 2) on wheat plant growth under salt stress. Inoculation with this bacterium can regulate the nutrient enrichment and Na^+^/K^+^ ratio of plant rhizosphere soil, thereby protecting the plant from salt damage. Therefore, our report expands our understanding of the plant growth-promoting properties of the genus *Bacillus*, especially *Bacillus subtilis*, which is highly salt-tolerant.

In conclusion, the rhizoplane of plants in saline-alkali land may be the reservoir of abundant PGPB, and rhizoplane bacteria may have the potential to promote plant growth and colonize the rhizosphere soil and plant interior. *Bacillus subtilis* HG-15 isolated from the rhizoplane of wheat in the Yellow River delta showed prominent salt tolerance and PGP activity in soils under no-salt stress, low salt stress, and high salt stress. At the same time, the strain was able to colonize the rhizosphere soil and the root, stem, and leaf surfaces of wheat. The chemical properties of wheat rhizosphere soil were significantly correlated with plant osmotic regulation, oxidation resistance, photosynthesis, and biomass accumulation. The effect of PGPB on rhizosphere soil is closely related to plant growth.

## Figures and Tables

**Table 1:** Biochemical and physiological characteristics of HG-15.

Characteristic (s)	Activity	Carbohydrate	Utilization	Carbohydrate	Utilization	Chemical sensitivity assays	Reaction
Gram stain	+	*α*-Cyclodextrin	+	Glycerol	+	Troleandomycin	−
Glucose oxidative fermentation	Fermentation type	Dextrin	+	*γ*-Aminobutyric acid	+	Rifamycin SV	−
Aerobic test	Facultative anaerobic	Glycogen	+	L-serine	+	Minocycline	−
Catalase test	+	D-mannitol	+	D-serine	+	Lincomycin	−
Nitrate reduction	−	D-melibiose	+	L-phenylalanine	+	Guanidine HCl	−
Starch hydrolysis	+	D-psicose	+	L-ornithine	+	Vancomycin	−
V-P test	+	Acetic acid	+	Hydroxy-L-proline	+	Tetrazolium violet	−
Salt tolerance 5% NaCl	+	Formic acid	+	Bromosuccinic acid	+	Tetrazolium blue	−
Salt tolerance 10% NaCl	+	Succinamic acid	+	Citric acid	+	Nalidixic acid	−
Citrate assimilation test	+	L-leucine	−	L-histidine	−	Sodium bromate	−
Methyl red test	−	L-fucose	−	Uridine	−	Potassium tellurite	+
Phenylalanine amino acid deaminase	+	*α*-D-lactose	−	L-threonine	−	1% sodium lactate	+

+ positive, − negative.

**Table 2:** Plant growth-promoting effects of the *Bacillus subtilis* HG-15 strain on wheat traits.

Plant growth-promoting traits	Activity
ACC deaminase production	14.816 ± 0.965 *μ*mol/(mg·h)
1AA production	154.53 ± 4.17 *μ*g/mL
ABA production	0.609 ± 0.026 *μ*g/mL
GA_3_ production	0.103 ± 0.005 *μ*g/mL
ZA production	0.638 ± 0.014 *μ*g/mL
JA production	0.430 ± 0.016 *μ*g/mL
SA production	3.865 ± 0.098 ng/mL
Proline production	19.862 ± 0.4748 *μ*g/mL
Glutamic acid production	33.488 ± 1.066 mg/mL
GSH production	0.187 ± 0.006 *μ*mol/mL
Siderophore production	+
Ammonia production	13.577 ± 0.530 *μ*g/mL
Nitrogen fixation	24.304 ± 0.7536 mg N/g glucose
Potassium solubilizing	+
Xylanase	76.813 ± 2.097 *u*/mL
*VOCs*	2,3-Butanediol, [R-(R∗,R∗)]-/2,3-butanediol/2-heptanone/-nonanone/2-nonanol/2-dodecanone/pentadecane/heptadecane

Notes: values are mean values ± standard deviations. Noninoculated bacteria were used as a negative control. + positive, − negative.

**Table 3:** Population densities reflecting wheat rhizosphere colonization by the HG-15 strain in soils with different salt concentrations (10^6^ CFU·g^−1^ dry weight of soil).

Salinity conditions	Days after inoculation (d)			
	7	14	21	28
0.15%	31.40 ± 2.99a	25.36 ± 2.39b	4.20 ± 1.57c	2.38 ± 0.72c
0.25%	9.50 ± 1.61a	6.37 ± 1.60b	0.76 ± 0.15c	0.57 ± 0.05c
0.35%	4.41 ± 1.65a	1.65 ± 0.72b	0.22 ± 0.08b	0.15 ± 0.02b

Notes: 0.15%, 0.25%, and 0.35% represent the soil with different salt concentrations used in the pot experiment. Values are expressed as the mean ± standard deviation (*n* = 3). Statistical analysis was accomplished with one-way ANOVA followed by Duncan's test. Means sharing a common letter within the same column are not significantly different at *p* < 0.05.

**(a):** 

Group	pH		EC		Organic matter		Olsen-P		Total N		Available N		Na	
	*F*	*p*	*F*	*p*	*F*	*p*	*F*	*p*	*F*	*p*	*F*	*p*	*F*	*p*
HG-15	68.535	≤ 0.001^∗∗^	145.997	≤0.001^∗∗^	908.604	≤0.001^∗∗^	6.017	0.030^∗^	167.186	≤0.001^∗∗^	48.793	≤0.001^∗∗^	33.947	≤0.001^∗∗^
Salt	15.305	≤0.001^∗∗^	6316.708	≤0.001^∗∗^	308.999	≤0.001^∗∗^	3.817	0.052	162.139	≤0.001^∗∗^	82.217	≤0.001^∗∗^	266.133	≤0.001^∗∗^
Salt × HG − 15	4.078	**0.045** ^∗^	24.481	**≤0.001** ^∗∗^	75.668	**≤0.001** ^∗∗^	0.517	0.609	12.810	**0.001** ^∗∗^	2.913	0.093	2.537	0.120

**(b):** 

Group	K		Ca		Mg		Dry weight		Fresh weight		Plant height		Root length	
	*F*	*p*	*F*	*p*	*F*	*p*	*F*	*p*	*F*	*p*	*F*	*p*	*F*	*p*
HG-15	17.668	0.001^∗∗^	7.003	0.021^∗^	47.294	≤0.001^∗∗^	213.390	≤0.001^∗∗^	65.135	≤0.001^∗∗^	213.390	≤0.001^∗∗^	65.135	≤0.001^∗∗^
Salt	21.402	≤0.001^∗∗^	11.735	0.001^∗∗^	25.205	≤0.001^∗∗^	155.056	≤0.001^∗∗^	72.974	≤0.001^∗∗^	155.056	≤0.001^∗∗^	72.974	≤0.001^∗∗^
Salt × HG − 15	1.415	0.281	0.168	0.847	1.018	0.391	14.222	**0.001** ^∗∗^	4.887	**0.028** ^∗^	14.222	**0.001** ^∗∗^	4.887	**0.028** ^∗^

**(c):** 

Group	*A*		*gsw*		*E*		*Ci*		Chl a		Chl b		*Fv/Fm*	
	*F*	*p*	*F*	*p*	*F*	*p*	*F*	*p*	*F*	*p*	*F*	*p*	*F*	*p*
HG-15	11.917	0.005^∗∗^	29.032	≤0.001^∗∗^	19.478	0.001^∗∗^	2.268	0.158	25.887	≤0.001^∗∗^	12.366	0.004^∗∗^	2.354	0.151
Salt	33.375	≤0.001^∗∗^	66.938	≤0.001^∗∗^	96.001	≤0.001^∗∗^	10.053	0.003^∗∗^	36.902	≤0.001^∗∗^	33.775	≤0.001^∗∗^	23.968	≤0.001^∗∗^
Salt × HG − 15	0.835	0.458	5.049	**0.026** ^∗^	1.663	0.230	0.901	0.432	5.425	**0.021** ^∗^	0.491	0.624	0.622	0.553

**(d):** 

Group	Proline		TSS		Total protein		MDA		POD		SOD		CAT	
	*F*	*p*	*F*	*p*	*F*	*p*	*F*	*p*	*F*	*p*	*F*	*p*	*F*	*p*
HG-15	273.491	≤0.001^∗∗^	198.372	≤0.001^∗∗^	171.471	≤0.001^∗∗^	327.578	≤0.001^∗∗^	7.205	0.020^∗^	0.829	0.380	10.479	0.007^∗∗^
Salt	449.485	≤0.001^∗∗^	117.815	≤0.001^∗∗^	112.776	≤0.001^∗∗^	140.613	≤0.001^∗∗^	91.949	≤0.001^∗∗^	21.897	≤0.001^∗∗^	338.937	≤0.001^∗∗^
Salt × HG − 15	104.857	**≤0.001** ^∗∗^	7.318	**0.008** ^∗∗^	4.670	**0.032** ^∗^	2.468	0.127	0.934	0.420	0.481	0.630	0.771	0.484

Notes: the interactive effect sizes of the bacterium and different soils on rhizosphere soil parameters and plant parameters based on two-way ANOVA with 95% confidence intervals. Data in the table are *p* values. ^∗^*p* < 0.05 and ^∗^*p* < 0.01. Dry weight: total weight of roots and shoots; fresh weight: total fresh weight of roots and shoots; *A*: net photosynthetic rate; *gsw*: stomatal conductance; *E*: transpiration rate; *Ci*: intercellular carbon dioxide concentration; Chl a: chlorophyll a; Chl b: chlorophyll b; TSS: soluble sugar; MDA: malondialdehyde; POD: peroxidase; SOD: superoxide dismutase; CAT: catalase. Bold indicates that there is a significant interaction between the bacterium and soil.

## Data Availability

All data generated or analyzed during this study are included in this published article.

## Supplementary Materials

Supplementary Materials Supplementary Materials S1: detailed description: phytohormone identification and quantification. Figure S1: BOX-PCR profile of bacteria colonizing wheat plants and confirmation of bacteria identity using pure culture. Figure S2: in vitro antagonistic activity of the *B. subtilis* HG-15 strain against selected pathogens in dual culture assays on PDA medium 72 h after incubation. Table S1: analysis “constrained” conditional term effects.
