# Supplementary material for: Bacillus subtilis HG-15, a Halotolerant Rhizoplane Bacterium, Promotes Growth and Salinity Tolerance in Wheat (Triticum aestivum)
Source: Biomed Res Int. 2022 May 7;2022:9506227. doi: 10.1155/2022/9506227 (PMC9107367; doi:10.1155/2022/9506227)
Supplement: Supplementary Materials — S1: detailed description: phytohormone identification and quantification. Figure S1: BOX-PCR profile of bacteria colonizing wheat plants and confirmation of bacteria identity using pure culture. Figure S2: in vitro antagonistic activity of the B. subtilis HG-15 strain against selected pathogens in dual culture assays on PDA medium 72 h after incubation. Table S1: analysis “constrained” conditional term effects. [file 9506227.f1.docx]

Supplementary Text

MATERIALS AND METHODS

Detailed description: phytohormone identification and quantification

Bacterial cultures (NFb) in the exponential growth phase were separated into several 20 mL fractions for identification of abscisic acid (ABA), zeatin (ZA), salicylic acid (SA), jasmonic acid (JA), and gibberellin 3 (GA3). The ABA HPLC liquid phase conditions were as follows: mobile phase A was methanol, whereas mobile phase B was 1% aqueous acetic acid in an isocratic elution (50% A + 50% B). ZA HPLC liquid phase conditions were as follows: mobile phase – mobile phase A was methanol, whereas mobile phase B was water in an isocratic elution (30% A + 70% B). The SA HPLC liquid phase conditions were as follows: mobile phase–mobile phase A was methanol, whereas mobile phase B was 1% aqueous acetic acid in an isocratic elution (60% A + 40% B). JA HPLC liquid phase conditions were as follows: mobile phase – mobile phase A was acetonitrile, whereas mobile phase B was 0.1% aqueous phosphate in an isocratic elution (60% A + 40% B). The GA3 HPLC liquid phase conditions were as follows: mobile phase – mobile phase A was methanol, whereas mobile phase B was 1% aqueous acetic acid in an isocratic elution (35% A + 65% B). ABA, ZA, SA, JA, and GA3 were determined using the corresponding HPLC method at UV wavelengths of 254, 254, 294, 210, and 254 nm.


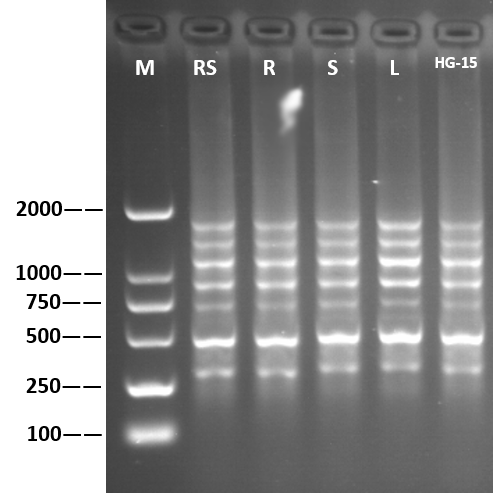
**Figure S1.** BOX-PCR profile of bacteria colonizing wheat plants and confirmation of bacteria identity using pure culture. Lane M: DNA marker, Lane RS: DNA of *B. subtilis* isolated from rhizosphere soil of inoculated plants, Lane R: DNA of *B. subtilis* isolated from roots of inoculated plants, Lane S: DNA of *B. subtilis* isolated from rhizosphere stems of inoculated plants, Lane L: DNA of *B. subtilis* isolated from leaves of inoculated plants, Lane HG-15: control DNA.


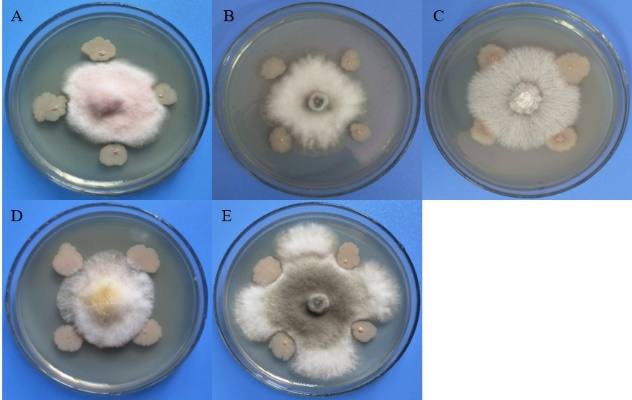


**Figure S2.** In vitro antagonistic activity of the *B. subtilis* HG-15 strain against selected pathogens in dual culture assays on PDA medium 72h after incubation. (A) *Fusarium oxysporum*, (B) *Fusarium pseudograminearum*, (C) *Rhizoctonia solani*, (D) *Fusarium graminearum*, (E) *Botryosphaeria ribis*.

**Table S1 | Analysis 'constrained' conditional term effects**

| Name | Explains % | pseudo-*F* | *p* |
| --- | --- | --- | --- |
| Na | 76.6 | 52.3 | 0.002 |
| OM | 4.6 | 3.7 | 0.004 |
| EC | 3.2 | 2.9 | 0.006 |
| Ca | 2.2 | 2.1 | 0.034 |
| Mg | 1.8 | 1.8 | 0.068 |
| TN | 2.3 | 2.8 | 0.028 |
| K | 0.7 | 0.8 | 0.536 |
| AP | 0.6 | 0.7 | 0.570 |
| pH | 0.7 | 0.7 | 0.600 |
| AN | 0.4 | 0.4 | 0.862 |
